# Determining the prognostic significance of alternative splicing events in soft tissue sarcoma using data from The Cancer Genome Atlas

**DOI:** 10.1186/s12967-019-2029-6

**Published:** 2019-08-23

**Authors:** Xia Yang, Wen-ting Huang, Rong-quan He, Jie Ma, Peng Lin, Zu-cheng Xie, Fu-chao Ma, Gang Chen

**Affiliations:** 1 Department of Pathology, First Affiliated Hospital of Guangxi Medical University, 6 Shuangyong Road, Nanning, 530021 Guangxi Zhuang Autonomous Region People’s Republic of China; 2 Department of Medical Oncology, First Affiliated Hospital of Guangxi Medical University, 6 Shuangyong Road, Nanning, 530021 Guangxi Zhuang Autonomous Region People’s Republic of China; 3 Department of Medical Ultrasonics, First Affiliated Hospital of Guangxi Medical University, 6 Shuangyong Road, Nanning, 530021 Guangxi Zhuang Autonomous Region People’s Republic of China

**Keywords:** Soft tissue sarcomas, STS, Alternative splicing, Splicing factors, Prognosis, TCGA

## Abstract

**Background:**

Surgery, adjuvant chemotherapy, and radiotherapy are the primary treatment options for soft tissue sarcomas (STSs). However, identifying ways to improve the prognosis of patients with STS remains a considerable challenge. Evidence shows that the dysregulation of alternative splicing (AS) events is involved in tumor pathogenesis and progression. The present study objective was to identify survival-associated AS events that could serve as prognostic biomarkers and potentially serve as tumor-selective STS drug targets.

**Methods:**

STS-specific ‘percent spliced in’ (PSI) values for splicing events in 206 STS samples were downloaded from The Cancer Genome Atlas SpliceSeq^®^ database. Prognostic analyses were performed on seven types of AS events to determine their prognostic value in STS patients, for which prediction models were constructed with the risk score formula $\sum\nolimits_{i}^{n} {PSIi\; *\;\beta i}$. Prediction models were also constructed to determine the prognostic value of AS events, and Spearman’s rank correlation coefficients were calculated to determine the degree of correlation between splicing factor expression and the PSI values.

**Results:**

A total 10,439 events were found to significantly correlate with patient survival rates. The area under the time-dependent receiver operating characteristic curve for the prognostic predictor of STS overall survival was 0.826. Notably, the splicing events of certain STS key genes were significantly associated with STS 2-year overall survival in the present study, including exon skip (ES) events in MDM2 and EWSR1, alternate terminator events in CDKN2A and HMGA2 for dedifferentiated liposarcoma, ES in MDM2 and alternate promoter events in CDKN2A for leiomyosarcoma, and ES in EWSR1 for undifferentiated pleomorphic sarcoma. Moreover, splicing correlation networks between AS events and splicing factors revealed that almost all of the AS events showed negatively correlations with the expression of splicing factors.

**Conclusion:**

An in-depth analysis of alternative RNA splicing could provide new insights into the mechanisms of STS oncogenesis and the potential for novel approaches to this type of cancer therapy.

## Background

Soft tissue sarcomas (STSs), which arise predominantly from the embryonic mesoderm, are a group of rare malignancies with high histological heterogeneity within each subtype [1]. STSs are found in almost every part of the human body, including the trunk, retroperitoneum, and the head and neck [2–4], commonly presenting as a symptomless mass. STS is associated with a morbidity of approximately 1.28/100,000 to 1.72/100,000, accounting for 0.73–0.81% of all malignancies [5] and 6% of childhood cancers [6]. Five-year overall survival (OS) with STS was reported to be approximately 50% [7].

More than 50 separate histologic STS subtypes have been identified, and the most common are liposarcoma (LPS), leiomyosarcoma (LMS), undifferentiated pleomorphic sarcoma (UPS), malignant peripheral nerve sheath tumors, and synovial sarcoma [5]. Proper STS diagnosis and treatment are always challenging for physicians and pathologists due to its extremely low incidence and the variable biological behavior among subtypes. The optimal management of heterogeneous malignancy is synergistic and mainly depends on the tumor’s location, size, and grade. Surgical resection remains the most effective method of curing early STS but is less successful in the treatment of advanced STS [2, 5]. Further studies are warranted to elucidate the molecular characteristics of these tumors, and the identification of additional diagnostic markers would be extremely beneficial in the clinical management of all STS patients.

Previously, researchers have focused on exploring effective diagnostic or prognostic markers in STSs using genomic data, such as gene expression [8], copy number variation [9], and DNA methylation [10]. However, changes in the transcript architecture that occur as a result of alternative splicing (AS) have largely been ignored. Many studies have demonstrated that the dysregulation of AS events is involved in tumor pathogenesis and progression [11–15]. AS occurs at a fundamental regulatory crossroad between transcription and translation that is conducive to creating protein diversity in mammals [16]. Up to 95% of highly-evolved eukaryotic species, especially human multi-exon genes, have been shown to produce multiple isoforms through AS [17, 18]. More specifically, AS, which takes place in a limited number of genes in the human genome, is thought to play a major role in increasing the functional complexity and diversity of proteins. Furthermore, AS is significantly involved in the homeostatic regulation of cells [19]. Aberrant splicing, which can lead to pathologic conditions, such as cancer, may be induced by the action of tumor suppressors or the mutation of oncogene splicing factors, thereby influencing cancer-related pathways [20, 21]. Moreover, the dysregulation of splicing behavior has been found to be associated with mutations or abnormal splicing factor expression [22, 23].

To date, a few studies have reported that AS is involved in various disease states, including cancerous malignancy (e.g., lung [24], ovarian [25], colorectal [12], and bladder carcinomas [26], as well as several gastrointestinal adenocarcinomas [27]). It appears that AS events are quite common in oncogenesis. However, the systematic analysis of AS, including the comprehensive genome-wide profiling of STS patients, has not been performed. New opportunities to evaluate cancer transcriptomes in relatively large populations have developed following dynamic advances in next-generation sequencing technology [28]. Moreover, it is now possible to identify unknown transcripts and splicing isoforms using RNA-seq, in addition to acquiring the computable measurement of alternatively-spliced protein variants, which can then be tested for associations with cancer [11].

Thus, an attempt was made in the present study to determine the association between AS events and the survival rate of STS patients using relevant data from The Cancer Genome Atlas (TCGA) database. More importantly, the objectives were to identify survival-associated AS events that could serve as prognostic biomarkers, which could then be targeted by tumor-selective STS drugs.

## Materials and methods

### ‘Percent spliced in’ for each AS event type

STS-specific percent spliced in (PSI) values for the splice events inferred from TCGA STS samples were downloaded from TCGA SpliceSeq^®^ (http://bioinformatics.mdanderson.org/TCGASpliceSeq), an AS database created by applying SpliceSeq^®^ analysis methods to RNA-seq samples [29]. The PSI value, which is in the range of 0 to 1 for a splicing event, is the ratio of normalized read counts that signify the insertion of a transcript component to the total normalized reads for a particular event [29]. In total, seven different types of splice events were downloaded, including alternate acceptors (AAs), alternate donors (ADs), alternate promoters (APs), alternate terminators (ATs), exon skips (ESs), mutually exclusive exons (MEs), and retained introns (RIs).

### Survival analysis in relation to alternative splicing events in The Cancer Genome Atlas soft tissue sarcoma cohort

The clinical characteristics of the STS cohort were downloaded from the TCGA data portal (https://tcga-data.nci.nih.gov/tcga/). Data corresponding to histologic subtypes with more than 30 samples and patients with at least 90 days of OS were included for further analysis. Finally, 206 samples comprising three histologic subtypes (57 dedifferentiated LPS, 100 LMS, and 49 UPS) were included in this study.

### The construction of prognostic prediction models

A univariate Cox regression was first applied to calculate the association between AS events and OS using R/Bioconductor^®^ (version 3.4.2). Then, the most significantly differentiated splicing events (i.e., the top 10) for each histologic subtype identified using univariate Cox regression were further subjected to multivariate Cox regression using IBM SPSS^®^ Statistics 22 (IBM Corporation, Armonk, NY) for the purpose of screening for independent factors predictive of STS. Lastly, prediction models were built with significant splicing events using multivariate Cox regression. The sample cohorts were divided into high- and low-risk groups according to the median PSI value. The prediction models for STS OS were constructed with the formula

$${\text{Risk score}} = \mathop \sum \limits_{i}^{n} PSIi\;*\;\beta i$$

where n represents the number of splicing events contained in the prediction model, i represents a certain splicing event, and β represents the regression coefficient.

To visualize the prognostic value of AS events, time-dependent receiver-operator characteristic (ROC) curves (estimated using the censored data) were created using the ‘survivalROC’ package in R for each model, and the ‘ggplot2’ and ‘survminer’ packages were also applied in R to generate a survival curve for each model. All the reported p-values were two-sided. An UpSet plot, a novel technique employed in the quantitative analysis of interactive sets, was used to present visualizations of the intersections between the seven types of AS events via the ‘UpSetR’ package in R [30].

### RNA extraction and polymerase chain reaction (PCR) validation

To verify that AS events occurring in soft tissue sarcoma are not occasional cases, three STS survival-associated AS events (MDM2_22969_ES, MFF_57799_ES, and CD74_74077_ES) were detected in STS samples in house. Two fresh STS samples were collected from the First Affiliated Hospital of Guangxi Medical University. Then, total RNA was extracted using an AxyPrep Multisource Total RNA Miniprep Kit (AXYGEN), followed by reverse transcription into cDNA using a MiScript^®^ II RT SuperMix Kit. According to splicing information in the TCGASpliceSeq database, the PSI values of MDM2_22969_ES in most of the STS samples was 1, and MDM2_22969_ES event occurred in the 10th and 11th exon skips; while both of the mean PSI values of MFF_57799_ES and CD74_74077_ES were 0.3, MFF_57799_ES event occurred in the 9th exon skip, and CD74_74077_ES event occurred in the 8th exon skip. Hence, the primers used to validate the three AS events were separately designed as follows. First, for MDM2_22969_ES, the forward primer (primer 1) was designed in the 9th exon as 5′-ATTCAGATGAATTATCTGGTGAACG-3′, and the reverse primer (primer 2) was designed in the 12th exon 5′-TGAGTTTTCCAGTTTGGCTTTCT-3′; Then, for MFF_57799_ES, the forward primer (primer 3) was designed in the 8th exon (5′-AAGGTTCCAGGCACCGATTT-3′), and the reverse primer (primer 4) in the 11th exon (5′-GCTGCATCTACAACAGTCAGG-3′). Finally, for CD74_74077_ES, the forward primer (primer 5) was designed in the 7.1th exon, and the reverse primer (primer 6) was designed in the 9th exon (forward primer: 5′-GCACCATTGGCTCCTGTTTG-3′; reverse primer: 5′-AGAAGACGGGTCCTCCAGTT-3′). The PCR system contained 10 μl of 2× PCR master mix (Thermofisher) and 1 μl of the forward and reverse primers, respectively, as well as 1 μl of cDNA and 7 μl Nuclease-Free Water, up to a total volume of 20 μl for the reaction system. Cycling conditions for PCR were 95 °C for 3 min, followed by 35 cycles at 95 °C for 0.5 min, 60 °C for 0.5 min, and 72 °C for 1 min. Finally, PCR products were sized by electrophoresis on 2% agarose gel. If the predictions in the TCGASpliceSeq database are accurate, the AS event does not occur, or the PSI value of the AS event is 1 (i.e., the AS event occurred in all mRNAs of the target gene), the PCR product should show a single band. Otherwise, the PCR product should contain two bands.

### Using The Cancer Genome Atlas soft tissue sarcoma cohort to identify potential relationships between AS events and genetic alterations in several genes

To explore how AS events occur and their role in STS, we preliminarily examined genetic alterations in MDM2, EWSR1, CDKN2A, and HMGA2, the four key genes involved in STS, and then assessed their correlation with AS events. First, the cBio Cancer Genomics Portal (cBioPortal), an open platform for exploring multidimensional cancer genomics data (http://www.cbioportal.org/) [31], was utilized to assess for gene copy number alteration (CNA) and mutation. The methylation levels of MDM2, EWSR1, CDKN2A, and HMGA2 were also calculated by comparing patterns in 265 STS samples and 4 normal controls obtained from the UCSC Xena Public Data Hub (https://xenabrowser.net/datapages/). Then, the mRNA levels of these four genes were calculated from TCGA RNA-seq data, Genotype-Tissue Expression project RNA-seq data, and Gene Expression Omnibus microarray data. All samples were pooled by conducting meta-analyses. Before performing the meta-analyses, the ‘SVA’ package for R was used to remove the batch effect so that data generated with the same platform could be merged into one dataset. Furthermore, a Spearman’s rank correlation test was performed to assess the degree of correlation between AS events and genetic alterations in MDM2, EWSR1, CDKN2A, and HMGA2 using R.

### The creation of correlation networks

To further investigate the relationship between splicing events and splicing factors, 66 splicing factors were downloaded from the SpliceAid^®^ database (http://193.206.120.249/splicing_tissue.html) [32]. The expression profiles of the splicing factors (level 3 mRNA-seq data) were also downloaded from TCGA. Spearman’s rank correlation coefficients were calculated to evaluate the degree of correlation between splicing factor expression and the PSI values of survival-associated AS. A p-value < 0.05 was considered statistically significant. Cytoscape^®^ (version 3.6.0) was applied to construct the correlation plots.

## Results

### A comprehensive analysis of AS events in the soft tissue sarcoma cohort

**Fig. 1:**
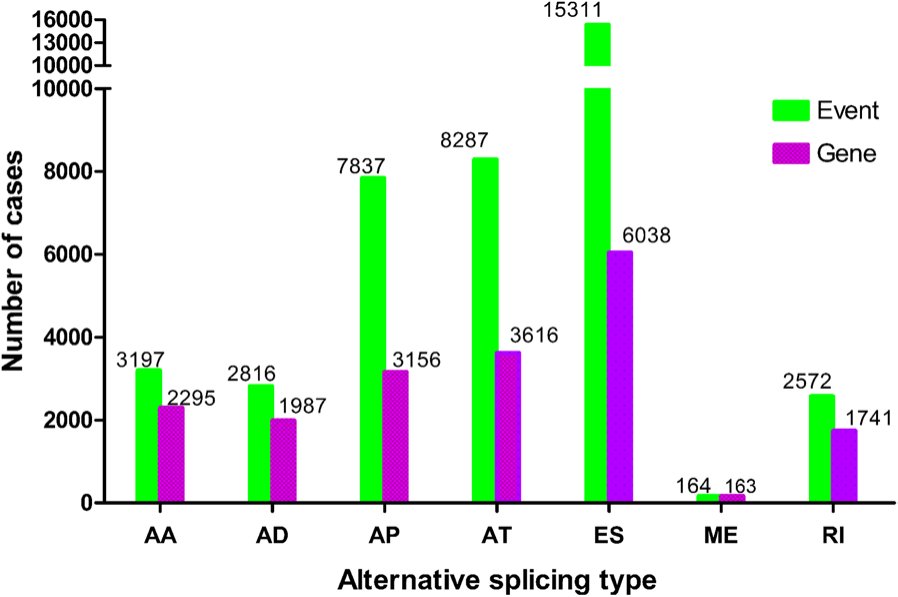
The number of alternative splicing events and involved genes for the 206 soft tissue sarcoma patients. ES is the most frequent of the seven types of events. *AA* alternate acceptor, *AD* alternate donor, *AP* alternate promoter, *AT* alternate terminator, *ES* exon skip, *ME* mutually exclusive exon, *RI* retained intron

Splicing events were comprehensively analyzed for 206 STS patients (57 dedifferentiated LPS patients, 100 LMS patients, and 49 UPS patients) based on relevant TCGA data. In total, 40,184 AS events were detected in 3064 genes, comprising 15,311 ES events in 6038 genes, 8287 AT events in 3616 genes, 7837 AP events in 3156 genes, 2572 RI events in 1741 genes, 3197 AA events in 2295 genes, 2816 AD events in 1987 genes, and 164 ME events in 163 genes (Fig. 1). Only one type of AS event was detected in most genes, although there were some exceptions; generally, it was demonstrated that 2–3 splicing events could be attributed to one gene, with a maximum of 5 types of AS events observable for a single gene. However, ES was the predominant type of event in all the histologic STS subtypes, which revealed that ES was the most common splicing event in STS.

### Survival-associated AS events in dedifferentiated liposarcoma, leiomyosarcoma, and undifferentiated pleomorphic sarcoma cohorts

**Fig. 2:**
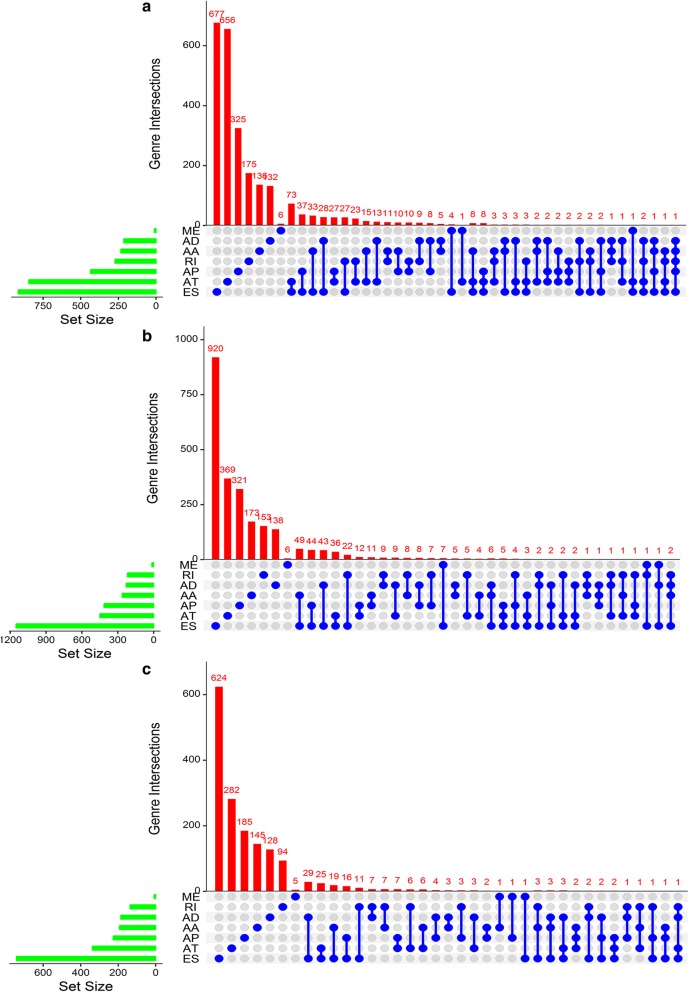
UpSet plots of alternative splicing events in the three histologic soft tissue sarcoma subtypes. **a**–**c** UpSet plots of interactions between the seven types of survival-associated alternative splicing events and genes. In this study, up to five types of alternative splicing associated with patient survival can be attributed to one gene. **a** Dedifferentiated liposarcoma. **b** Leiomyosarcoma. **c** Undifferentiated pleomorphic sarcoma cohorts

**Fig. 3:**
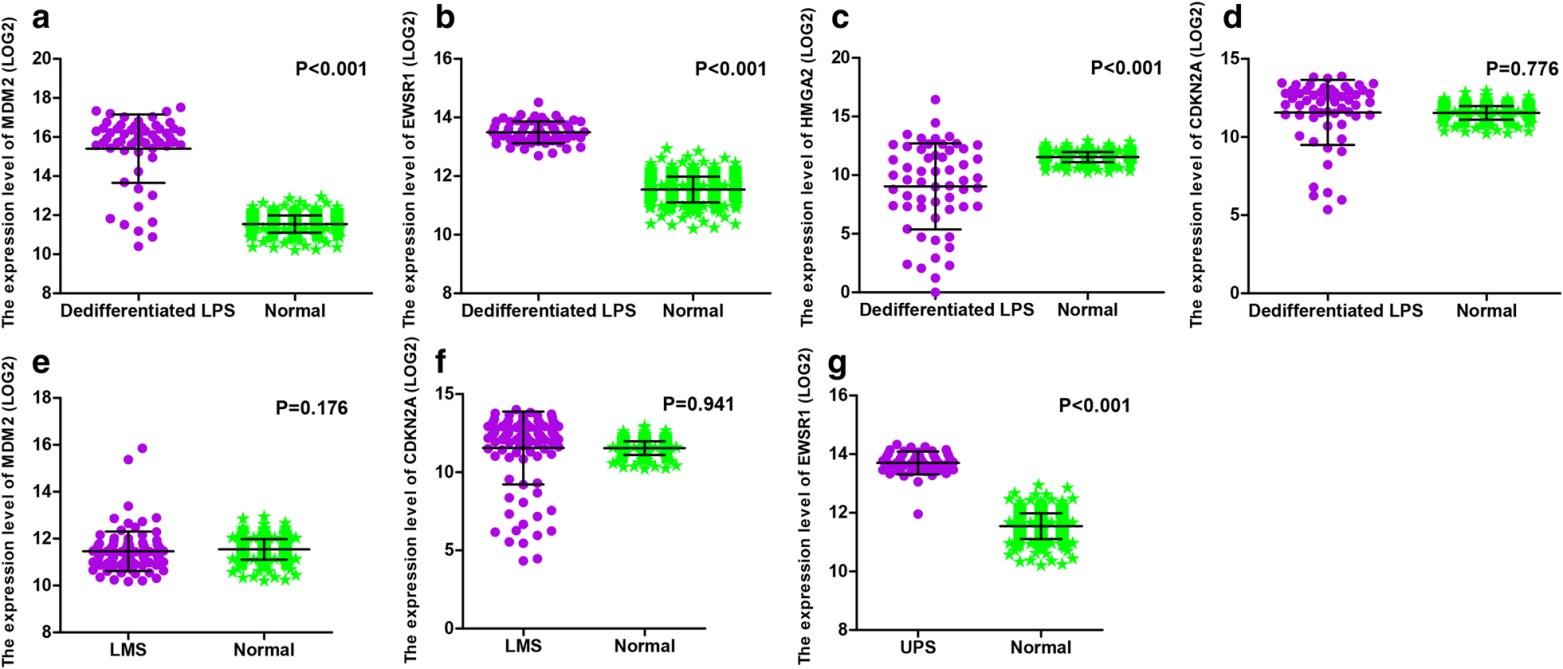
The expression of *MDM2*, *EWSR1*, *CDKN2A*, and *HMGA2* in soft tissue sarcoma. **a**–**d** mRNA levels of *MDM2*, *EWSR1*, *CDKN2A*, and *HMGA2* in dedifferentiated LPS and normal controls. **e**, **f** mRNA levels of *MDM2* and *CDKN2A* in LMS and normal controls. **g** mRNA levels of *EWSR1* in UPS and normal controls. *Dedifferentiated LPS* dedifferentiated liposarcoma, *LMS* leiomyosarcoma, *UPS* undifferentiated pleomorphic sarcoma

**Fig. 4:**
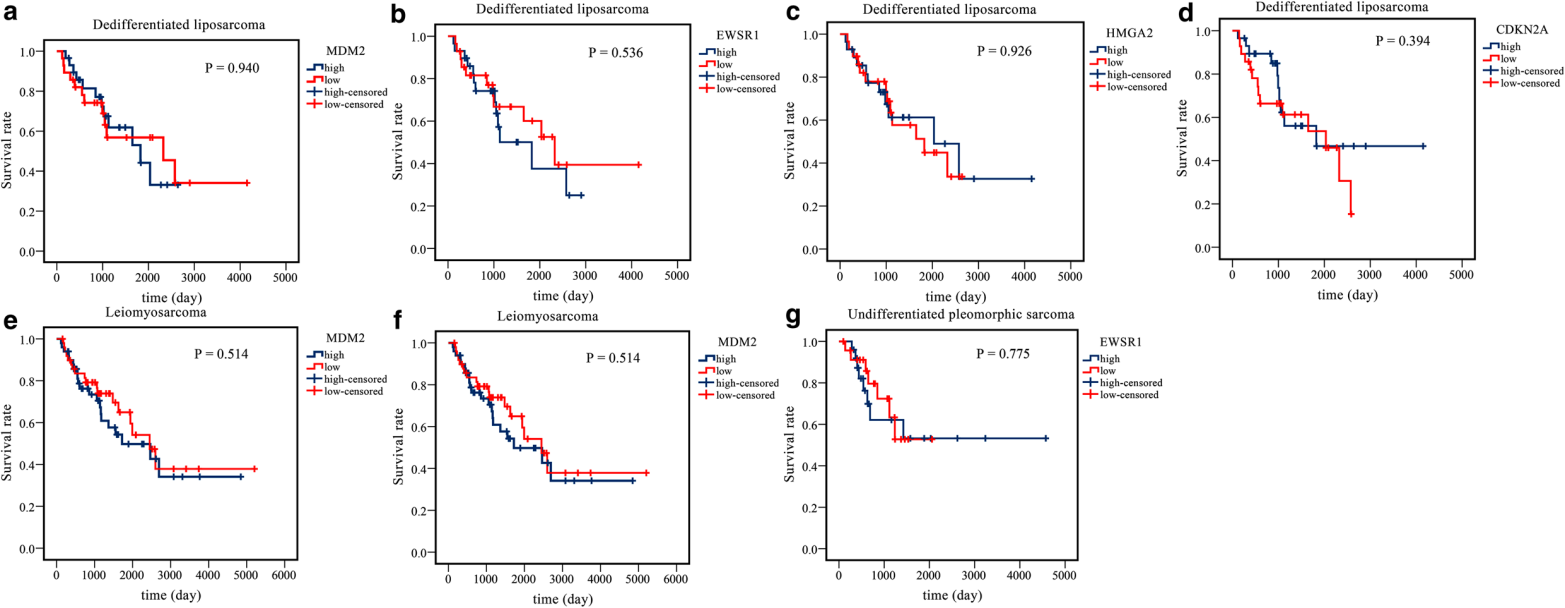
The prognostic values of *MDM2*, *EWSR1*, *CDKN2A*, and *HMGA2* in soft tissue sarcoma. **a**–**d** The prognostic values of *MDM2*, *EWSR1*, *CDKN2A*, and *HMGA2* in dedifferentiated liposarcoma. **e**, **f** The prognostic values of *MDM2* and *CDKN2A* in leiomyosarcoma. **g** The prognostic value of *EWSR1* in undifferentiated pleomorphic sarcoma. No significant differences were found between the gene high-expression group and the low-expression group in the three types of soft tissue sarcoma

Univariate survival analysis was conducted to evaluate the association between AS events and OS in the dedifferentiated LPS, LMS, and UPS cohorts. A total of 4471, 3672, and 2381 survival-associated AS events were detected in the dedifferentiated LPS, LMS, and UPS cohorts, respectively (p < 0.05). An UpSet plot was generated to visualize significant survival-associated AS events (Fig. 2a–c). Notably, of the significant prognostic AS events, patient survival in the dedifferentiated LPS cohort was observed to be associated with five events for MRPL55 (i.e., AA, AD, AP, ES, and RI in MRPL55). MRPL55 is one of the mitoribosome-specific proteins, which are reported to play an important role in the regulation of cell death and act upon tumor suppressors [33]. Accordingly, AS events may result in the inactivation of MRPL55 functioning. Furthermore, ES in MDM2 and EWSR1 and AT in CDKN2A and HMGA2 for dedifferentiated LPS, ES in MDM2 and AP in CDKN2A for LMS, and ES in EWSR1 for UPS were also shown to be significant survival-associated AS events in the present study. However, analyzing the mRNA levels of MDM2, EWSR1, CDKN2A, and HMGA2 in dedifferentiated LPS, MDM2 and CDKN2A in LMS, and EWSR1 in UPS revealed that MDM2 and EWSR1 were upregulated in dedifferentiated LPS, while HMGA2 was downregulated in dedifferentiated LPS, and EWSR1 was upregulated in UPS, but none of them showed any prognostic value in the three histologic STS subtypes (Figs. 3 and 4).

### Confirmation of three AS events using clinical samples

**Fig. 5:**
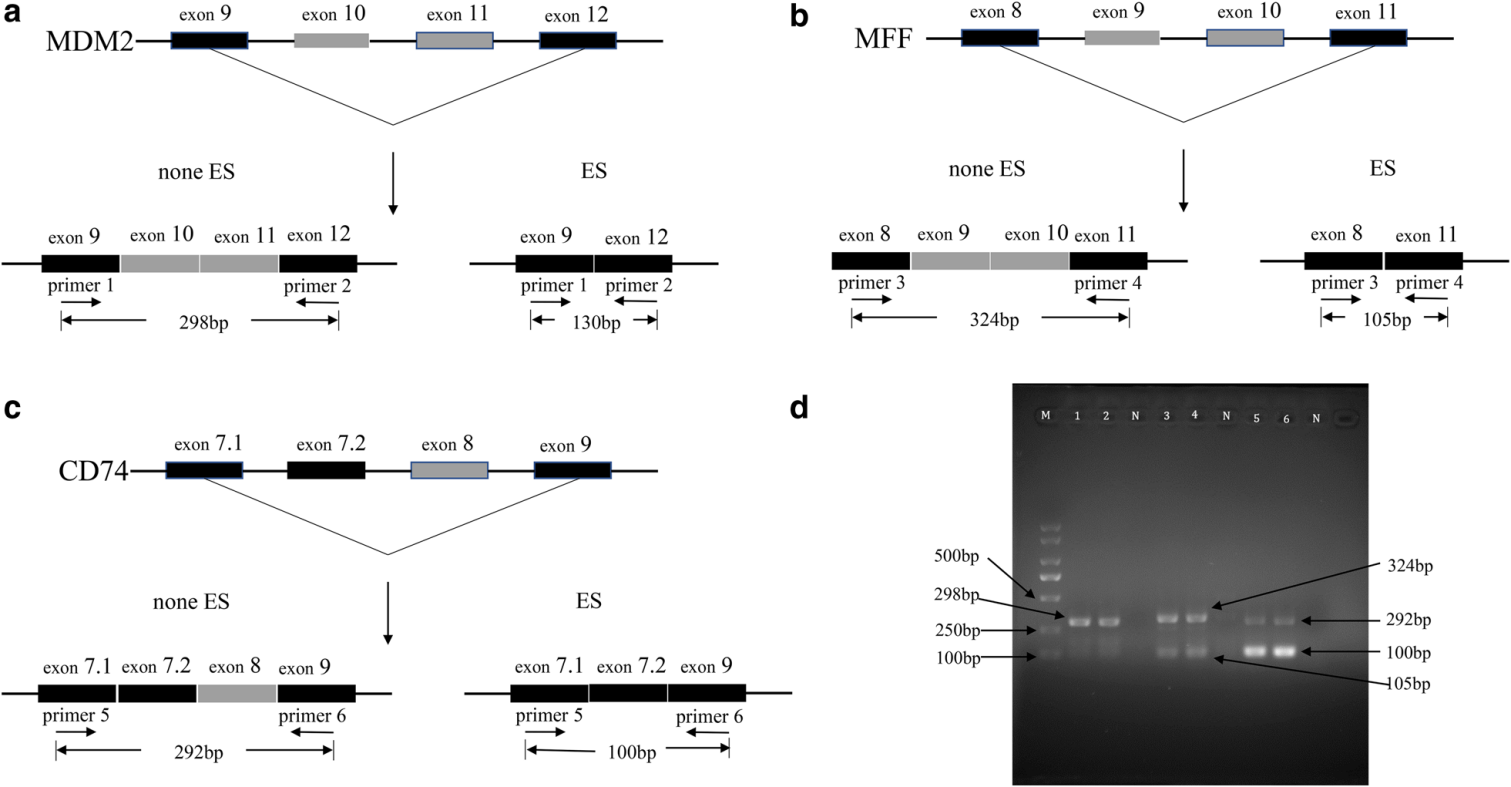
Three ES events in STS samples validated via polymerase chain reaction. **a**–**c** A schematic diagram of MDM2_22969_ES, MFF_57798_ES, and CD74_ 74077_ES events. The ES events of MDM2, MFF, and CD74 occurred in exons 10 and 11, exon 9, and exon 8, respectively. To validate these events, upstream and downstream primers were designed on either side of the skipping exons. If the ES event did not occur, all the exons would have been retained; electrophoresis produced a single band, and the size of the PCR product was the maximum. Otherwise, the skipping exon was deleted and produced another band. **d** An electropherogram of the three ES events. M represents a DNA marker; N represents the negative control without a template. Lanes 1 and 2 are products of the PCR amplification of MDM2_22969_ES events in two STS tissues; only a single band of 305 bp is found. Lanes 3 and 4 are products of the PCR amplification of MFF_57798_ES events in two STS tissues, and there are two bands of 324 bp and 105 bp. Lanes 5 and 6 are products of the PCR amplification of CD74_ 74077_ES events in two STS tissues; both 292 bp and 100 bp bands are found. *ES* Exon Skip, *STS* soft tissue sarcoma

By combining the TCGASpliceSeq database predictions and our PCR products in the STS samples, it could be easily found that MDM2_22969_ES only presented a single band in the STS samples, while both MFF_57799_ES and CD74_74077_ES presented two bands, which were in complete agreement with the predictions in the TCGASpliceSeq database (Fig. 5). The results revealed that the algorithm used for predicting AS events of tumors in the TCGASpliceSeq database is reliable.

### Genetic alterations and mRNA levels of MDM2, EWSR1, CDKN2A, and HMGA2 in soft tissue sarcomas

**Fig. 6:**
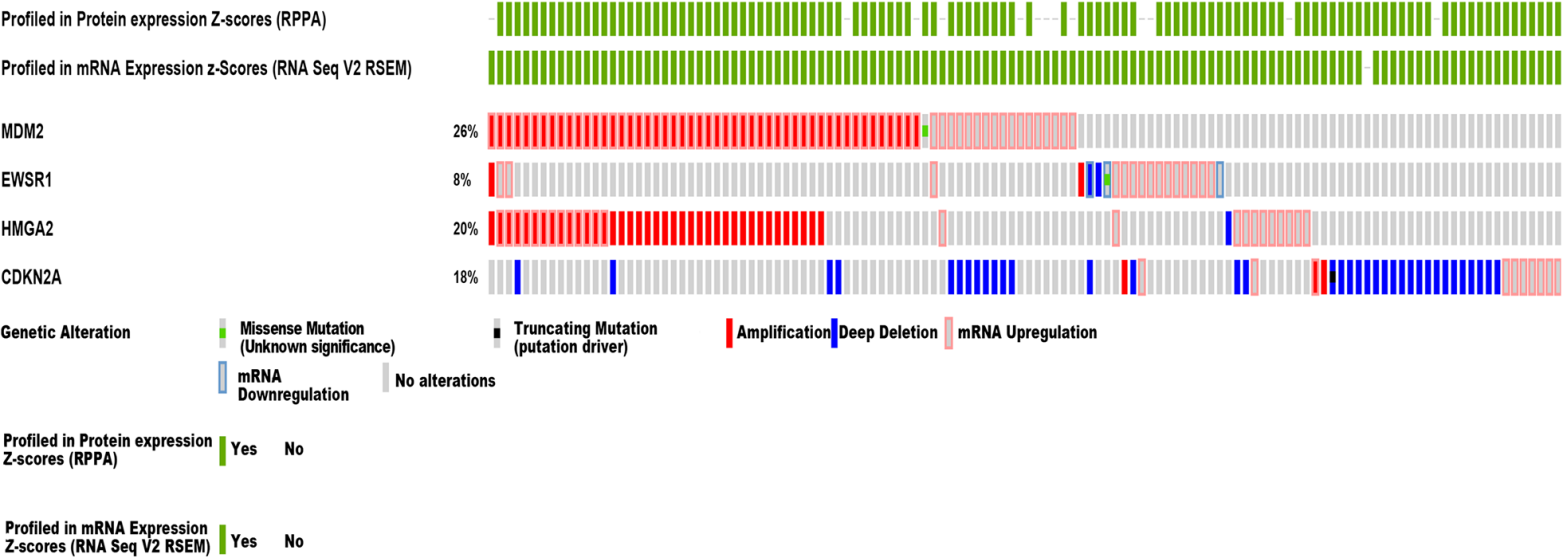
Genetic alterations of *MDM2*, *EWSR1*, *CDKN2A*, and *HMGA2* in soft tissue sarcoma. The main alteration type observed in *MDM2* and *HMGA2* was amplification, while the main alteration type observed in *CDKN2A* was deep deletion, and alterations of *EWSR1* were only found in a few soft tissue sarcoma cases

**Fig. 7:**
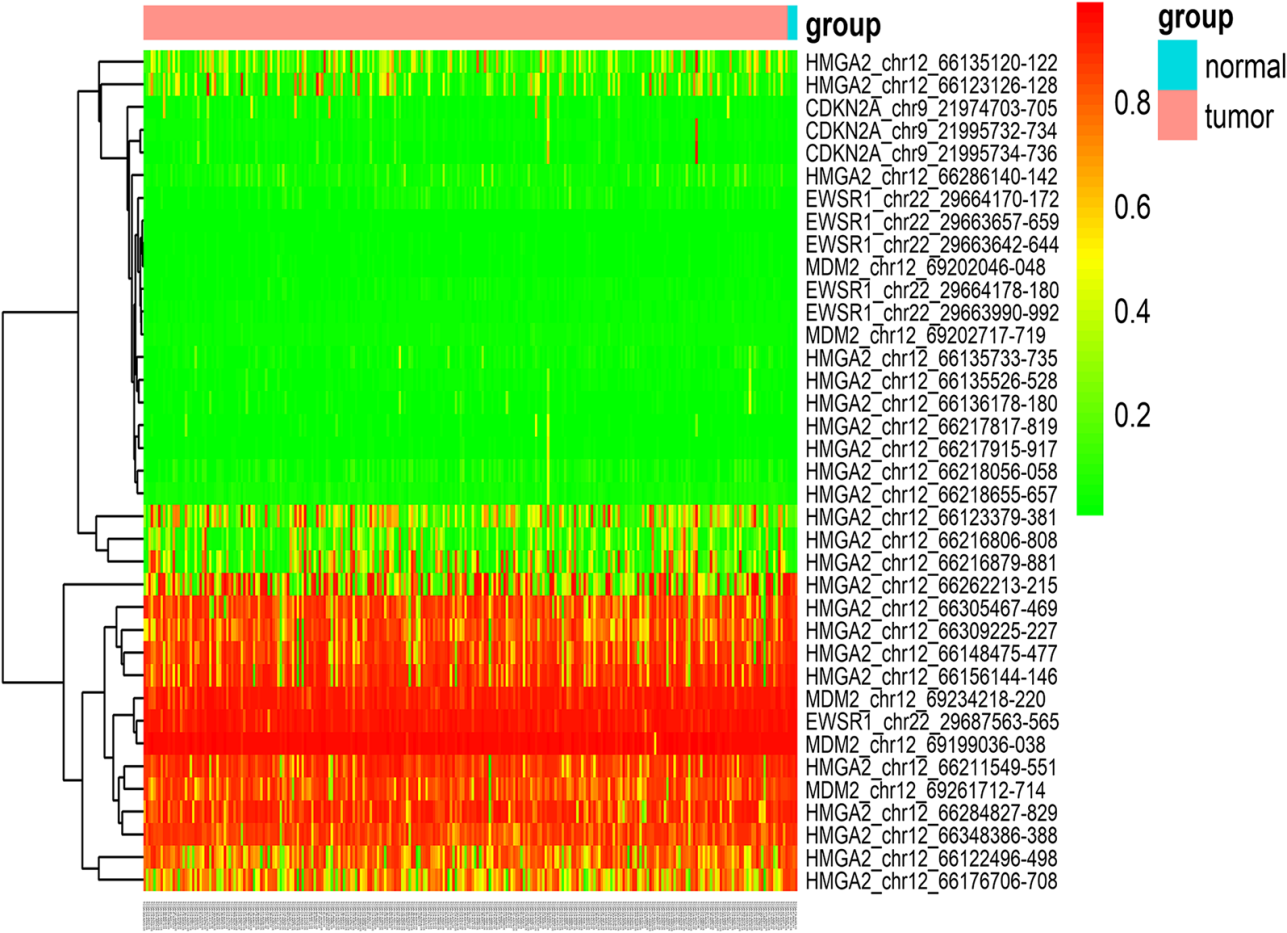
The methylation of *MDM2*, *EWSR1*, *CDKN2A*, and *HMGA2* in soft tissue sarcoma and normal controls. No obvious differences in methylation in these four genes were found between the tumor and normal control groups. The parts of the IDs represent the gene symbol, chromosome, methylation start site, and end site (gene_chrom_chromStart_chromEnd), respectively

**Fig. 8:**
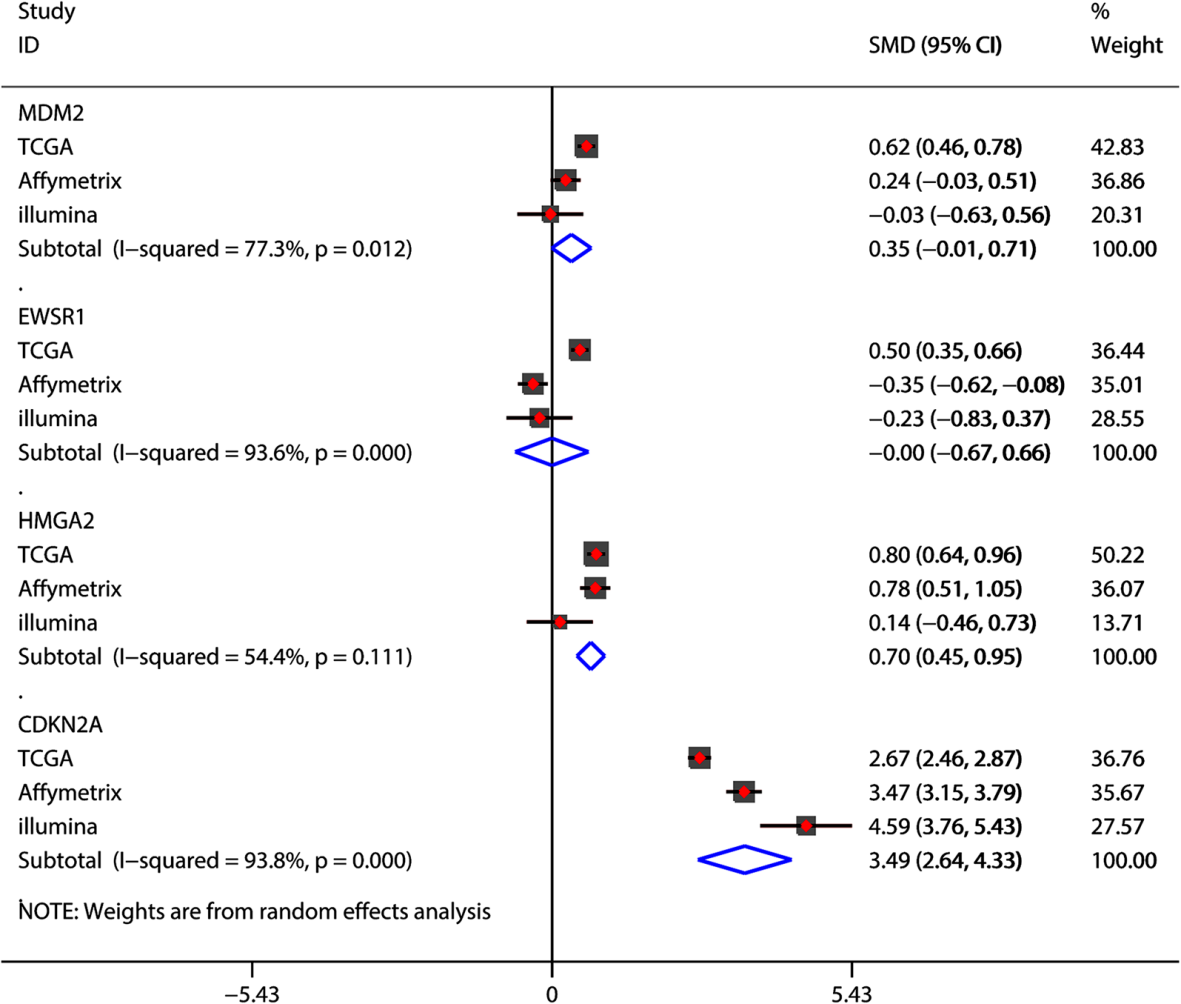
Meta-analyses conducted to evaluate the mRNA levels of *MDM2*, *EWSR1*, *CDKN2A*, and *HMGA2* in soft tissue sarcoma and normal controls. For RNA-Seq data, 257 STS samples were collected from TCGA, and 448 normal mesenchymal tissues from the GTEx database were chosen as normal controls. For the Affymetrix data, 57 STS samples and 730 normal controls were included. For the Illumina data, 12 STS samples and 111 normal controls were ultimately included. *SMD* standard mean difference, *95% CI* 95% confidence interval, *TCGA* The Cancer Genome Atlas, *GTEx* genotype-tissue expression project

**Fig. 9:**
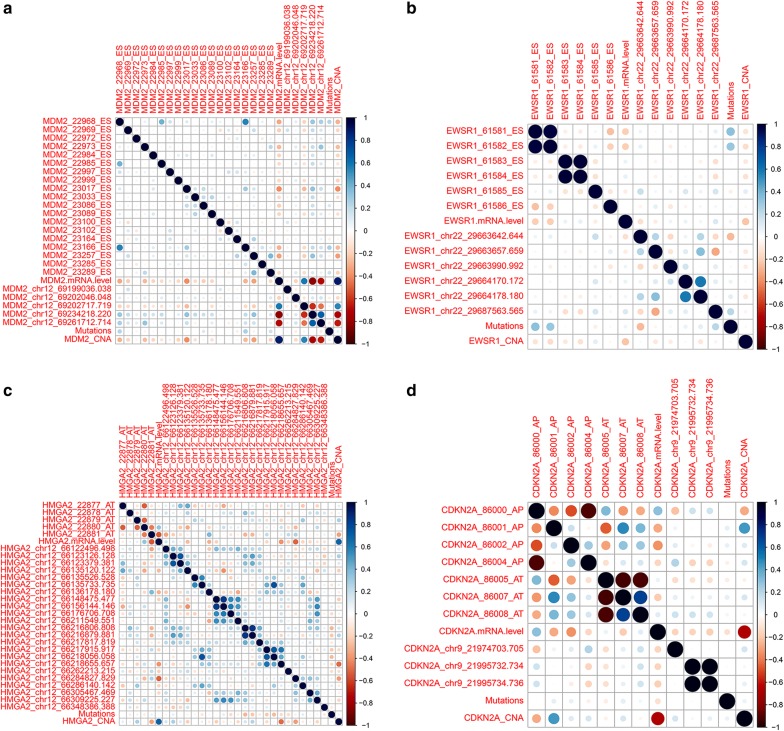
Spearman’s rank correlation tests evaluated the correlation between genetic alterations in *MDM2*, *EWSR1*, *CDKN2A*, and *HMGA2* and their AS events. **a** *MDM2*, **b** *EWSR1*, **c** *HMGA2*, **d** *CDKN2A*. Expected genetic alterations of *CDKN2A*, genetic alterations of another three genes showed relatively weak relationships with their AS events

The genes with prognostic splicing events were examined for a potential relationship with CNA, mutation, methylation, and AS events. MDM2, EWSR1, CDKN2A, and HMGA2 have been confirmed to exert crucial roles in STS tumorigenesis or progression. Therefore, these four genes were selected as examples. In the cBioPortal platform data, the most common alterations of MDM2 were amplifications, and there were a few missense mutations. For EWSR1, the alterations occurred only in a small number of cases, including amplification and deep deletion, as well as one case of missense mutation. The most frequent alterations in HMGA2 were amplifications. Events associated with CDKN2A included mostly deep deletion and a few amplifications, as well as one case of a truncation mutation (Fig. 6). The UCSC Xena Public Data Hub data revealed that MDM2, EWSR1, and HMGA2 showed high levels of DNA methylation in STS tissues (Fig. 7). The meta-analyses demonstrated that HMGA2 and CDKN2A levels were clearly upregulated when compared with normal controls, and MDM2 also showed a higher expression level in STS than in normal controls (Fig. 8). Spearman’s rank correlation tests revealed that, unlike the CNA of CDKN2A, the genetic alterations of the other three genes showed relatively weak relationships with their AS events (Fig. 9). However, although no statistically significant difference could be found between the mRNA levels of these four genes and their AS events, most of them presented a negative correlation with each other.

### The construction of prognostic models in the dedifferentiated liposarcoma, leiomyosarcoma, and undifferentiated pleomorphic sarcoma cohorts

The 10 most significant survival-associated AS events of all the splicing types (with the exception of ME events for UPS, for which there were < 10) were selected as potential prognostic factors for the three cohorts. Multivariate Cox regression was applied to eliminate any events that might not have independent prognostic value. Thereafter, separate prognostic models were constructed for the remaining AS events in all seven splicing types for the three cohorts.

**Fig. 10:**
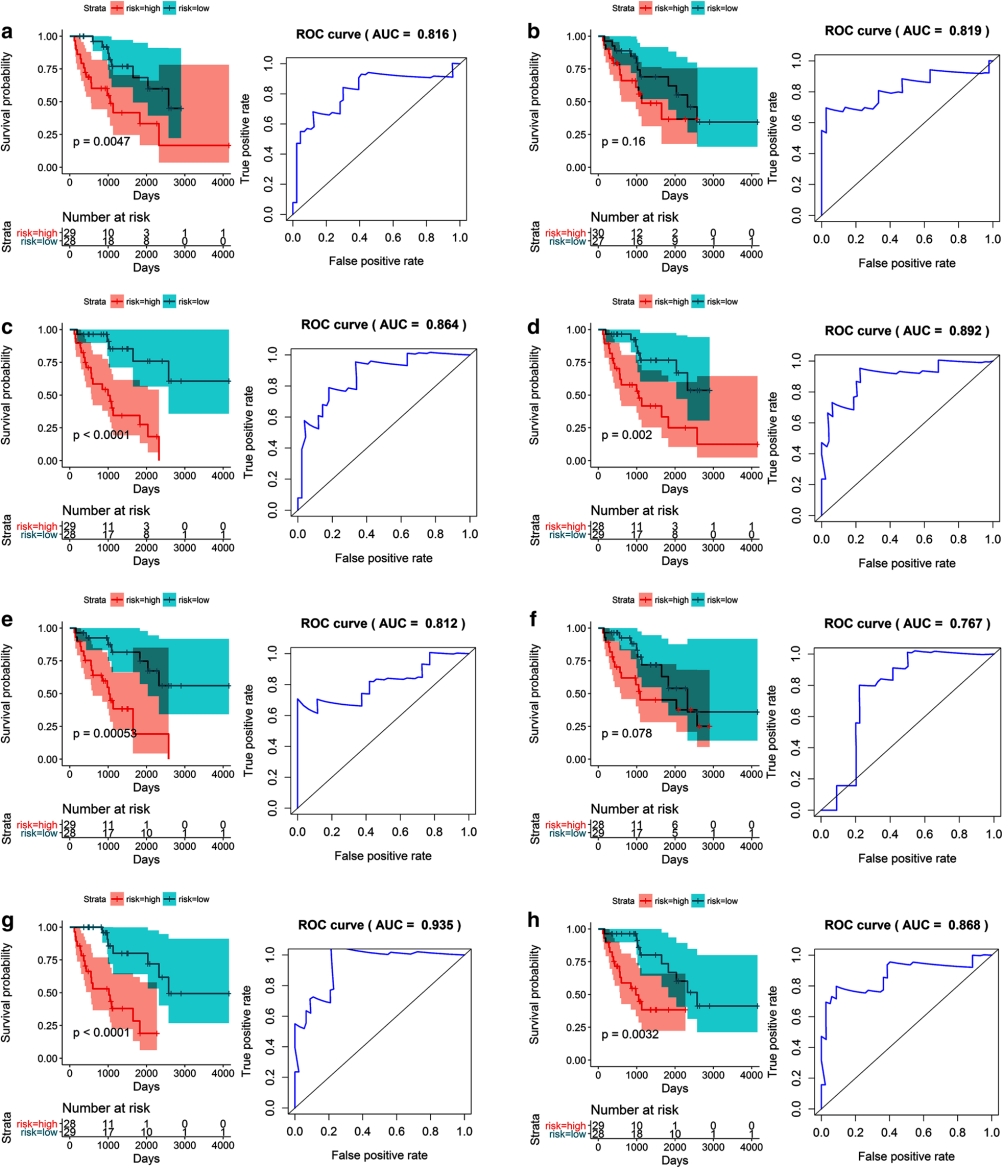
Kaplan–Meier (K–M) survival plots and receiving operating characteristic (ROC) curves of predictive factors in dedifferentiated liposarcoma. **a**–**g** K–M curves and ROC curves with AUCs for prognostic factors based on one type of alternative splicing in dedifferentiated liposarcoma patients (**a** AA, **b** AD, **c** AP, **d** AT, **e** ES, **f** ME, **g** RI). **h** A K–M curve and ROC curve with AUC for the final prognostic factors based on merged types of alternative splicing events in dedifferentiated liposarcoma patients. Prognostic models were constructed with the alternative splicing events that proved to be independent factors in the multivariate Cox regression analysis. *AA* alternate acceptor, *AD* alternate donor, *AP* alternate promoter, *AT* alternate terminator, *ES* exon skip, *ME* mutually exclusive exon, *RI* retained intron, *AUC* area under the curve, *K*–*M* Kaplan Meier, *ROC* receiver operating characteristic

With the exception of the AD and ME events, significantly different survival times were observed between the high- and low-risk groups in the dedifferentiated LPS cohort. The ROC curves confirmed that the prognostic model in which AP events were included had the maximum efficacy (AUC = 0.847) in distinguishing between good and poor survival prospects. Likewise, the prognostic model constructed to reflect the merged types of AS events also had superior prognostic value, with an AUC of 0.802 for 2-year survival (Table 1, Fig. 10).

**Table 1:** Prediction models for the dedifferentiated liposarcoma cohort based on each type of splicing event

Risk score	Model	HR (95% CI)	*p*-value	ROC
Risk score (AA)	COMT_61101_AA * 0.403 + EIF3C_35828_AA * (− 45.134) + USE1_48240_AA * (− 0.827)	3.316 (1.443–7.620)	0.0047	0.816
Risk score (AD)	CHCHD3_81837_AD * 8.254 + NUDT6_70526_AD * 0.188 + TAGLN_18897_AD * 0.330	1.817 (0.787–4.192)	0.16	0.819
Risk score (AP)	9-Sep_43720_AP * 1.240 + CD37_50911_AP * 0.147 + PAK1_17951_AP * 0.053	5.908 (2.545–13.72)	< 0.0001	0.864
Risk score (AT)	AIG1_77972_AT * (− 0.072) + BCAM_50346_AT * (− 0.107) + SATB1_63672_AT * (− 1.227)	3.664 (1.611–8.338)	0.002	0.892
Risk score (ES)	MANBAL_59341_ES * 3.744+ MAP4K4_54762_ES * 0.394 + TMEM107_39125_ES * 0.444	4.571 (1.935–10.80)	0.0005	0.812
Risk score (ME)	ARL6IP5_101550_ME * 0.146 + DDX42_42990_ME * 0.904 + DNM2_47585_ME * (− 0.511) + STK36_57557_ME * (− 0.156) + ZFAND6_32173_ME * (-3.154)	2.074 (0.922–4.666)	0.078	0.767
Risk score (RI)	GMFG_49768_RI * 0.169 + MRPL50_87090_RI * (− 1.882) + SLC25A35_39153_RI * (− 0.336) + SUGP2_48551_RI * 0.043	4.966 (1.993–12.37)	< 0.0001	0.935
Risk score (merged)	CHCHD3_81837_AD * 101.047 + NUDT6_70526_AD * 1.407 + TAGLN_18897_AD * 8.834 + MANBAL_59341_ES * 8.899 + MAP4K4_54762_ES * 1.304	3.770 (1.558–9.124)	0.0032	0.868

**Fig. 11:**
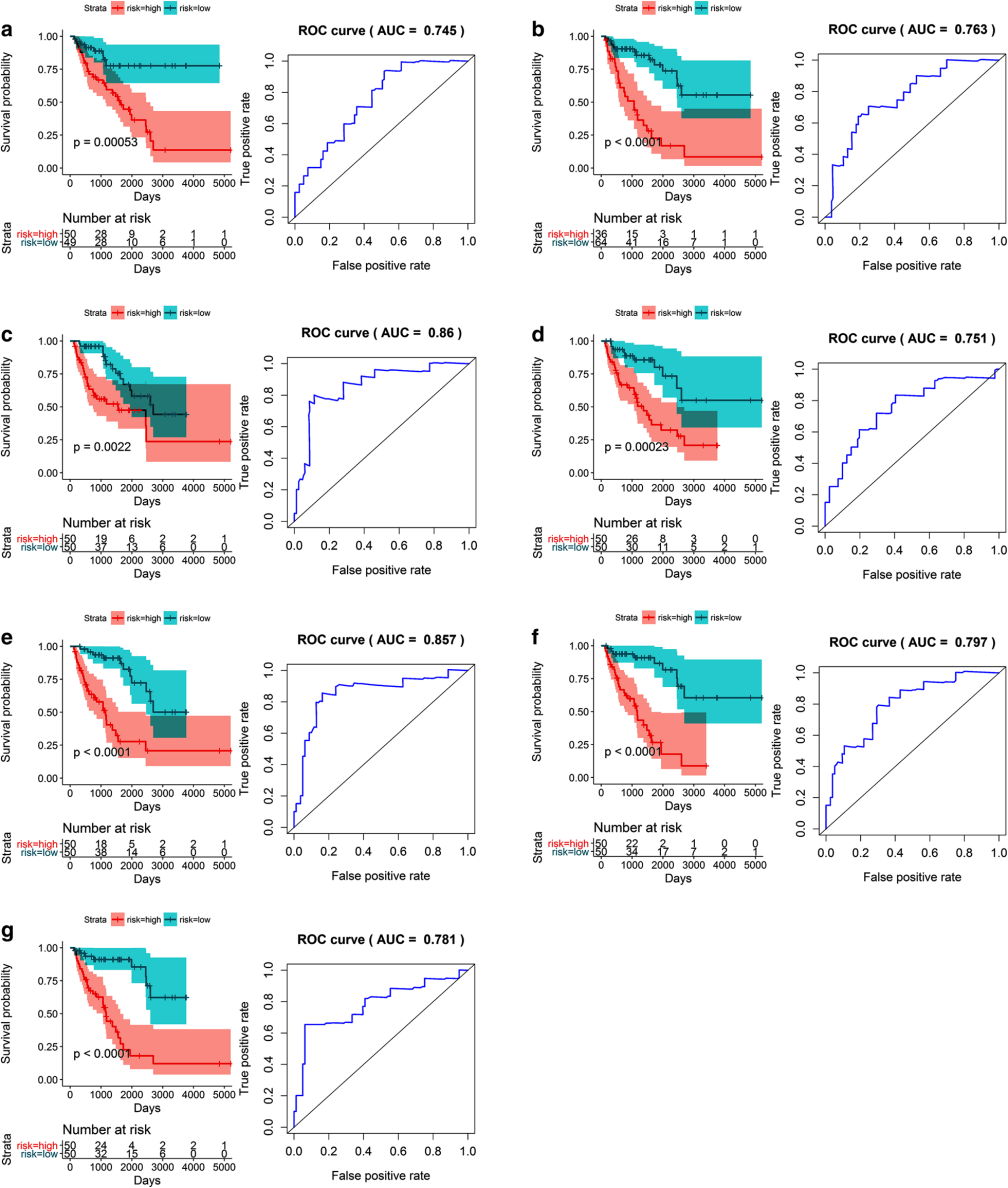
Kaplan–Meier (K–M) survival plots and receiving operating characteristic (ROC) curves of factors predictive of leiomyosarcoma. **a**–**f** K–M curves and ROC curves with AUCs in prognostic factors based on one type of alternative splicing event in leiomyosarcoma patients (**a** AA; **b** AD; **c** AP; **d** AT; **e** ES; **f** RI). An ME event could not be identified and, thus, could not be included in the analysis. **g** K–M curve and ROC curve with AUC of the final prognostic factors based on merged types of alternative splicing events in leiomyosarcoma. Prognostic models were constructed with the alternative splicing events that proved to be independent factors in the multivariate Cox regression analysis. *AA* alternate acceptor, *AD* alternate donor, *AP* alternate promoter, *AT* alternate terminator, *ES* exon skip, *ME* mutually exclusive exon, *RI* retained intron, *AUC* area under the curve, *K*–*M* Kaplan–Meier, *ROC* receiver operating characteristic

**Fig. 12:**
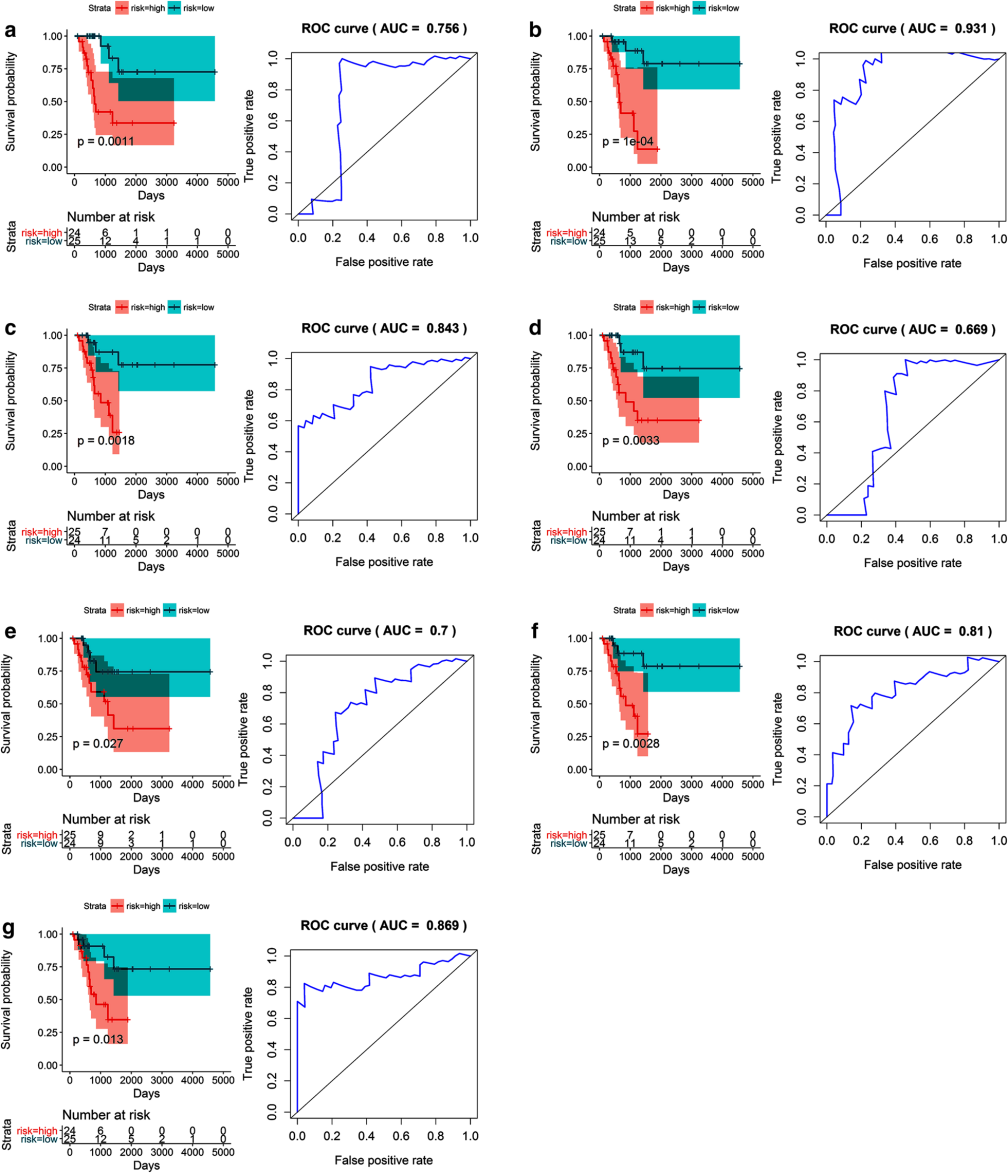
Kaplan–Meier (K–M) survival plots and receiver operating characteristic (ROC) curves of factors predictive of undifferentiated pleomorphic sarcoma. **a**–**f** K–M curves and ROC curves with AUCs of prognostic factors based on one type of alternative splicing event in undifferentiated pleomorphic sarcoma patients (**a** AD; **b** AP; **c** AT; **d** ES; **e** ME; **f** RI). An AA event was not available and, thus, could not be included in the analysis. **g** A K–M curve and ROC curve with AUC of the final prognostic factors based on merged types of alternative splicing events in undifferentiated pleomorphic sarcoma. Prognostic models were constructed with the alternative splicing events that proved to be independent factors in the multivariate Cox regression analysis. *AA* alternate acceptors, *AD* alternate donors, *AP* alternate promoters, *AT* alternate terminators, *ES* exon skips, *ME* mutually exclusive exon, *RI* retained intron, *AUC* area under the curve, *K*–*M* Kaplan–Meier, *ROC* receiver operating characteristic

Substantially varying survival times for the low- and high-risk patients in the LMS cohort were found to be associated with AS events. Of the eight prognostic models, the one that included AP events was the most accurate, with an AUC value for the ROC curve of 0.865 for 2-year survival. In contrast, the AUC value was 0.769 for 2-year survival based on the model created with the merged types of AS events (Table 2, Fig. 11). For the UPS cohort, the ineligibility of AA events precluded the necessity of building a model that included them, so six prognostic models were built based on specific events, and one was constructed using mixed events. Sound prognostic values were reported for all of these models, with the most accurate one including AD, AP, and AT events. The AUC for this prognostic model was the highest at up to 0.884 for 2-year survival, followed by the AP model with an AUC of 0.88 for 2-year survival (Table 3, Fig. 12).

**Table 2:** Prediction models for the leiomyosarcoma cohort based on each type of splicing event

Risk score	Model	HR (95% CI)	*p*-value	ROC
Risk score (AA)	COPA_8458_AA * (− 0.168) + DMTF1_80302_AA * 0.035 + SNRPN_29704_AA * 0.361	3.102 (1.635–5.886)	0.0005	0.745
Risk score (AD)	IFT81_24411_AD * 0.053 + NFATC4_26993_AD * (− 0.073) + RMI2_34011_AD * (− 0.092) + TLE3_31418_AD * (− 0.05)	6.875 (3.363–14.06)	< 0.0001	0.763
Risk score (AP)	CD2BP2_36095_AP * 0.463 + HMGCL_1078_AP * 0.228 + PAIP1_71959_AP * (− 0.209) + RAB5B_22326_AP * (− 0.175) + VAT1_41179_AP * 0.256	2.783 (1.445–5.360)	0.0022	0.86
Risk score (AT)	ABCA1_87106_AT * (− 0.429) + C12orf75_24135_AT * 3.806 + COA3_41129_AT * (− 0.334) + TMEM14B_75308_AT * 0.131	3.282 (1.743–6.180)	0.0002	0.751
Risk score (ES)	EDEM1_63033_ES * (− 0.51) + GNB2L1_75087_ES * (− 0.55) + NSUN5_270163_ES * 0.295 + RAB11FIP3_32895_ES * (− 0.089) + ZMYND11_10590_ES * (− 0.264)	5.290 (2.704–10.35)	< 0.0001	0.857
Risk score (RI)	CASP1_18519_RI * (− 0.043) + CCDC107_86266_RI * 0.103 + GBA2_86290_RI * (− 0.047) + KLHL25_32357_RI * (− 1.282) + TKT_65298_RI * (− 0.076)	6.271 (3.195–12.31)	< 0.0001	0.797
Risk score (merged)	NFATC4_26993_AD * (− 0.153) + HMGCL_1078_AP * 0.321 + COA3_41129_AT * (− 0.471) + TMEM14B_75308_AT * 0.328 + EDEM1_63033_ES * (− 0.447) + NSUN5_270163_ES * 0.493 + ZMYND11_10590_ES * (− 0.339)	5.678 (2.946–10.94)	< 0.0001	0.781

**Table 3:** Prediction models for the undifferentiated pleomorphic sarcoma cohort based on each type of splicing event

Risk score	Model	HR (95% CI)	*p*-value	ROC
Risk score (AD)	ATP6AP1_90605_AD * 2.273 + DNAJB12_12092_AD * (− 0.121) + KLC1_29490_AD * (− 0.111) + LRIF1_4128_AD * (− 0.28) + RAI14_71719_AD * (− 5.893)	5.742 (2.015–16.36)	0.0011	0.756
Risk score (AP)	6-Sep_89962_AP * (− 0.393) + ALKBH7_47027_AP * (− 0.255) + KIAA1217_10996_AP * 0.716 + UBR4_876_AP * 0.071	8.892 (2.951–26.80)	0.0001	0.931
Risk score (AT)	GNPDA1_73859_AT * (− 8.763) + IL18BP_17471_AT * 0.073 + KLHL26_48496_AT * 0.155	5.391 (1.877–15.49)	0.0018	0.843
Risk score (ES)	PARD3B_57099_ES * (− 0.448) + PDDC1_13757_ES * (− 1.314) + TMUB2_41797_ES * (− 0.193)	4.690 (1.674–13.14)	0.0033	0.669
Risk score (ME)	FYN_77273_ME * 0.128 + NDUFAF6_84594_ME * (− 0.059)	3.170 (1.143–8.788)	0.027	0.70
Risk score (RI)	ANKRD28_63628_RI * (− 0.917) + RAB43_66696_RI * (− 0.067) + ZNF773_52280_RI * 0.348	4.966 (1.739–14.18)	0.0028	0.81
Risk score (merged)	ATP6AP1_90605_AD * 16.015 + UBR4_876_AP * (− 0.25) + GNPDA1_73859_AT * (− 120.044) + KLHL26_48496_AT * 2.08	3.780 (1.324–10.79)	0.013	0.869

Among the three histologic subtypes of STS, AP events were the leading factors for predicting patients’ OS rates, which may indicate that AS events are likely to mostly be involved in the progression of STS and that tumor progression, in some patients, may involve the AP-type splicing of oncogenes or tumor suppressor genes. Additionally, predictive models created with the merged types of AS events had moderate to strong prognostic abilities for STS patients. Therefore, AS events may serve as promising markers for the prognosis of STS patients.

### The construction of prognostic models in the soft tissue sarcoma cohort

**Fig. 13:**
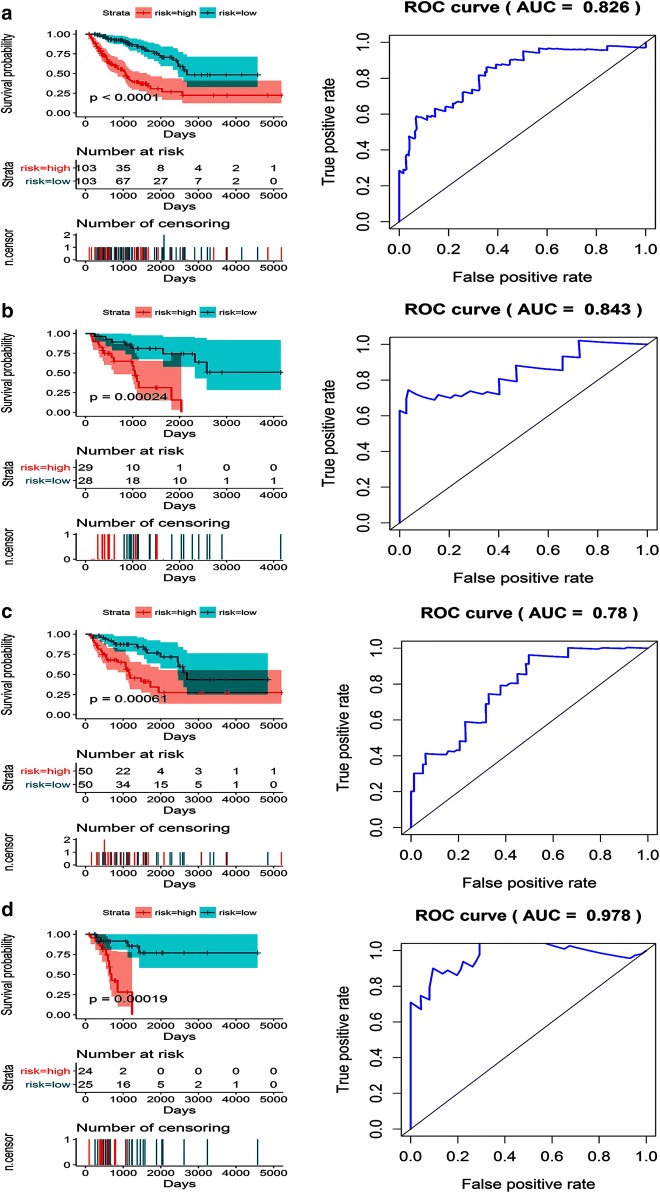
Kaplan–Meier (K–M) survival plots and receiver operating characteristic (ROC) curves of factors predictive of soft tissue sarcoma. **a** A K–M curve and ROC curve with AUC for factors predictive of STS. **b** A K–M curve and ROC curve with AUC for prognostic factors in the leiomyosarcoma subgroup. **c** A K–M curve and ROC curve with AUC for prognostic factors in the differentiated liposarcoma subgroup. **d** A K–M curve and ROC curve with AUC for prognostic factors in the undifferentiated pleomorphic sarcoma subgroup. *AUC* area under the curve, *K*–*M* Kaplan–Meier, *ROC* receiver operating characteristic, *STS* soft tissue sarcoma

Common survival-associated AS events in the dedifferentiated LPS, LMS, and UPS cohorts (p < 0.05) were screened for further survival analysis in the merged STS cohort to reduce the probability of misclassification, investigate similarities among the dedifferentiated LPS, LMS, and UPS cohorts, and identify prognostic factors that were applicable to STS patients. Notably, 26 AS events were eligible for a univariate Cox regression analysis in the STS samples. Following the multiple Cox regression analyses, 8 of the original 26 AS events remained for inclusion in a prognostic model (Risk score = LGALS3BP_43934_AA * 0.252 + RAMP2_41121_AP * 0.034 + GABRE_90380_AT * 0.051 + SDF4_39_AT * 0.280 + 11-Sep_69616_AT * 0.059 + IRAK1_90546_ES * (− 0.149) + CTNND1_15936_ES * (− 0.081) + NR1H3_15705_RI * 0.017). The predictiveness of survival for the models constructed from these eight events was sound for the STS cohort (HR, 4.111 [2.602–6.493], AUC = 0.826), dedifferentiated LPS cohort (HR [95% CI] 5.349 [2.183–13.11], ACU = 0.843) and the UPS cohort (HR [95% CI] 9.149 [2.862–29.25], AUC = 0.978), but less so for the LMS cohort (HR [95% CI] 3.165 [1.638–6.115], AUC = 0.780; Fig. 13). Although the prognostic accuracy for the STS cohort was less than that of the models for the dedifferentiated LPS and UPS cohorts (0.826 versus 0.868 and 0.869), the use of this model still has great potential in clinical practice.

### A correlation network of AS events and splicing factors

It is accepted that splicing occurs through the concerted actions of the multisubunit complex and is enhanced by splicing factors [34]. It has been reported in previous studies that AS events are influenced by the abnormal expression of splicing factors, which belong to the serine/arginine-rich (SR) family or heterogeneous nuclear ribonucleoproteins [35, 36]. When splicing occurs, the primary function of the splicing factors is to bind to the pre-mRNA regulatory sequences, facilitate splice site recognition, and promote the inclusion of alternatively-spliced exons and intronic and exonic splicing enhancers [37]. Thus, the present study further investigated whether the significantly different survival prospects associated with AS events were potentially regulated by specific splicing factors in the STS tissue. The expression profiles (level 3 RNA-seq data) of splicing factors in the STS cohort were downloaded from TCGA. A univariate Cox regression analysis demonstrated that 25, 12, and 6 splicing factors were significantly associated with survival in the dedifferentiated LPS, LMS, and UPS cohorts, respectively.

**Fig. 14:**
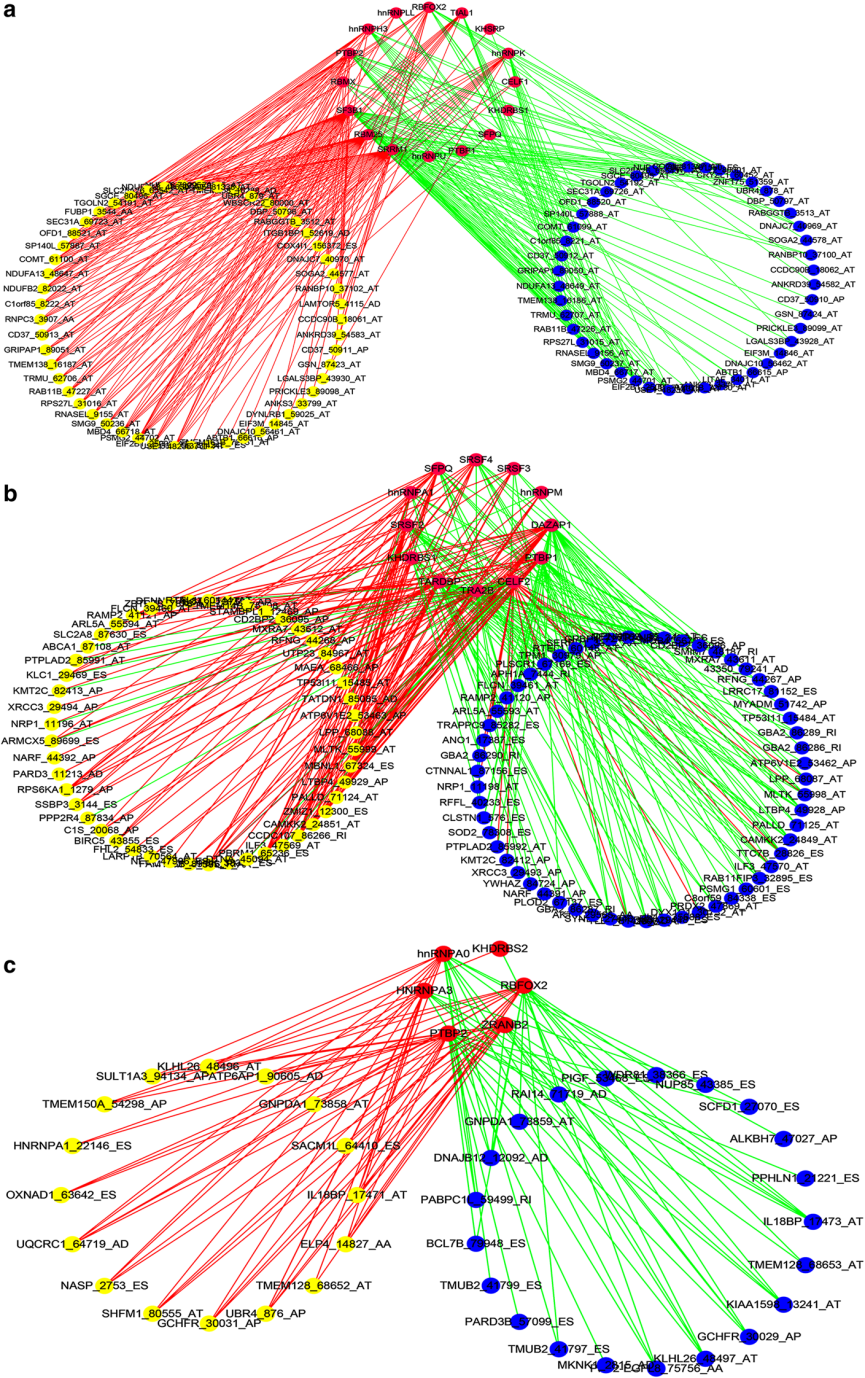
Survival-associated splicing factors and splicing correlation networks for three sarcoma subgroups. **a** Positive correlations (red lines) between 16 splicing factors (purple dots) and 162 alternative splicing (AS) events and negative correlations (green lines) between 16 splicing factors (purple dots) and 128 AS events in the dedifferentiated liposarcoma subgroup. **b** Positive correlations (red lines) between 12 splicing factors (purple dots) and 154 AS events and negative correlations (green lines) between 12 splicing factors (purple dots) and 166 AS events in the leiomyosarcoma subgroup. **c** Positive correlations (red lines) between 6 splicing factors (purple dots) and 16 AS events and negative correlations (green lines) between 6 splicing factors (purple dots) and 21 AS events in the undifferentiated pleomorphic sarcoma subgroup. AS events whose percent spliced in (PSI) values were positively or negatively correlated with survival times are represented by yellow or blue dots, respectively

A correlation analysis using Spearman’s rank correlation coefficient performed using splicing factors and survival-associated AS events found that, of the correlation between splicing events and factors in the dedifferentiated LPS cohort, there were 305 significant survival-associated AS events (p < 0.001) with 25 splicing factors included. The expression levels of 16 splicing factors were demonstrated to correlate positively and negatively with 162 and 128 AS events, respectively. The most significant correlation (p < 0.0001) is shown in Fig. 14a. In the LMS cohort, 297 significant survival-associated AS events (p < 0.001) and 12 splicing factors were considered. Of these, 154 survival-associated AS events were positively correlated with 12 splicing factors, and 166 survival-associated AS events were negatively correlated with 12 splicing factors. The correlation between the 297 significant survival-associated AS events and 12 splicing factors (p < 0.001) is highlighted in Fig. 14b. In the UPS cohort, the number of survival-associated AS events and factors was found to be 57 and 6, respectively. After performing the correlation analysis, 16 AS events were found to be positively correlated with 6 splicing factors, while 21 AS events were found to be negatively correlated with 6 splicing factors (Fig. 14c).

Notably, in the dedifferentiated LPS and UPS cohort, the AS events that were positively correlated (red lines) with the splicing factors were poor prognostic events (yellow dots), whereas the AS events that were negatively correlated (green lines) with the splicing factors were favorable (blue dots). In the LMS cohort, most of the AS events that were positively correlated (red lines) with the splicing factors were poor prognostic events (yellow dots). Likewise, most of the AS events that were negatively correlated (green lines) with the splicing factors were favorable prognostic events (blue dots).

Additionally, we attempted to investigate genetic alterations in 24 splicing factors and their association with splicing events using TCGA data. However, we found no clear evidence that mutations and the CNA of survival-associated splicing factors were correlated with AS events.

## Discussion

Identifying ways to improve the prognosis of patients with STS through the identification of factors predictive of the disease stage remains a considerable challenge. To date, STS treatment options are often limited to surgery, with the possibility of adjuvant chemotherapy and radiotherapy. However, traditional treatments kill cancer cells and damage normal cells, causing long-term developmental damage to the tissue. More research is needed to explore the potential molecular mechanisms of STS. Over the past two decades, increasing attention has been paid to the regulatory mechanisms of splicing thought to influence tumor development. The dysregulation of AS events has been reported to contribute to the pathogenesis of several kinds of tumors. This mechanism is, in turn, exploited by the cancer cells and reinforces their biological behavior (i.e., the promotion of cancer cell survival, metastasis, and drug resistance [13, 38, 39]). Studies of AS events might provide additional insight into the early diagnosis or prognosis evaluation, including the mechanisms behind STS genesis and development.

A fair number of studies have been conducted on splicing events. For example, CD44 is a cell surface molecule that has been shown, through AS events, to play a role in cancer. It has been found to be associated with breast cancer metastasis [40] and ovarian cancer metastasis with a poor prognosis [41], as well as participate significantly in melanoma progression [42], and it is a potential treatment target in colorectal cancer [43]. Other AS events in various genes, such as VEGFA, APP, and NUMB, have been reported to regulate the development of colorectal cancer and have demonstrated potential as new targets for the diagnosis, prognosis, and treatment of this type of malignancy [44]. Additionally, single-gene AS events were evaluated in STS by Jacob et al. [45]; their research irrefutably identified specific alternative splice variants of MDM2 and MDM4 as persistent biomarkers of rhabdomyosarcoma (RMS) tumors. They also demonstrated that MDM2-ALT1 could potentially serve as a prognostic marker for RMS metastasis, regardless of tumor histology. However, all of these studies were limited to the detection of specific genes or AS events in relation to a certain type of tumor. The occurrence and development of cancer is a process of complex and consecutive changes. Alternative splicing of genes would generate a large number of aberrant mRNA and protein isoforms with diverse regulatory and functional properties in cancer. Hence, integrating multiple AS events into an aggregated model could add more prognostic efficiency than single clinical indicators.

The development of high-throughput technology has catapulted the study of cancer biology transcriptomes into the digital era. Via this technology, AS events were found in various types of cancer and gradually caught the attention of scientists. TCGA SpliceSeq is a resource used for collecting AS events through high-throughput technology. Researchers have utilized this resource to identify potential prognostic AS events and potential treatment targets in several human cancers. However, integrating multiple AS events into an aggregated model to predict the prognosis of STS patients has not been reported. We used a small sample size of STS tissues to validate several AS events collected from the TCGA SpliceSeq database and found that the validated results were consistent with predicted data.

We constructed the first prognostic predictive models with AS events for STS patients using univariate Cox regression and multiple Cox regression analysis, which produced informative results. Clinical parameters (i.e., age, sex, and clinical stage) were also included in the univariate and multiple Cox regression analyses in our study. However, no clinical parameter was found to be an independent prognostic factor. According to our results, as well as those of previous studies focused on non-small cell lung cancers, bladder urothelial carcinoma, breast cancer, and other types of cancer [24, 26, 46], ES was the most common AS event regardless of tumor type, while ME events rarely occurred in cancer. This pattern was also reflected in the STS cohort and each of the three histologic subtypes of STS. However, an examination of AS events in specific genes showed varied associations between AS events and tumors. For example, we compared the prognostic splicing events of STS and bladder carcinoma evaluated by He et al. [26] and found that there was no overlap in prognostic splicing events for these two types of malignancies; even when the same genes exhibited the same AS events, they always occurred in different regions of the gene exons. In different histologic subtypes of STS, there were more overlapping AS events than in bladder carcinoma, but few AS events overlapped. The prognostic AS events presented distinct tissue specificity, which can be explained by tumors from different sources having different routes to pathogenesis, with each kind of tumor having its own traits and, hence, its own specific tumor markers. This unique characteristic of AS events suggests that survival-associated AS events could be used to construct accurate prognostic predictors for risk stratification in STS, which has promising implications in clinical practice.

According to the present study and other aforementioned studies, AS events do not occur randomly in STS, and most were found to be significantly correlated with STS patient survival times. It is likely that the occurrence of AS events activates oncogenes or inactivates tumor suppressors that affect the prognostic status of STS. With the development of technology, mutations within introns and synonymous mutations in exons have been gradually demonstrated to also affect gene function through AS events [47, 48]. Although our study did not provide sufficient evidence, we suspect that genetic alterations in STS oncogenes or tumor suppressors may affect the recognition of splicing sites by splicing factors, which, in turn, causes the activation of oncogenes or the inactivation of tumor suppressors.

Correlation analyses were also conducted in a preliminary exploration of the underlying mechanisms linking the expression of survival-associated splicing factors and AS events, and the results indicate that survival-associated splicing factors exert either a significant positive or negative regulatory effect on AS events. Almost all the favorable prognostic AS events were negatively correlated with splicing factors. Similarly, most of the poor prognostic AS events were positively correlated with splicing factors. This finding is consistent with those of previous reports [24, 49]. Hence, it is inferred that unfavorable STS-related prognoses may be attributed to a defect in the expression of specific splicing factors, thereby affecting normal splicing progress. However, since splicing events are regulated by splicing factors through the involvement of exonic or intronic regulatory sequences, related reports have indicated that DNA mutations [50], DNA methylation [51], and the aberrant histone modification of splicing factors [52] may influence their recognition of splicing sites, further leading to changes in splicing events. Overall, many uncertainties exist in how splicing factors regulate the occurrence of splicing events. The current study preliminarily explored their surface relationship, and therefore, more functional research is needed to uncover the underlying mechanism.

## Conclusion

We constructed several prognostic models for STS based on survival-associated AS events, which were shown to have high accuracy when applied to several of the most common types of STS samples and should, therefore, be considered for adoption in clinical practice. Moreover, correlation analyses between AS events and factors showed how splicing factors potentially regulate aberrant AS events. However, the current study has some limitations. First, it was based on a single data source (TCGA) without validation using other independent cohorts; we intend to remedy this limitation in the future with clinical samples. Second, STSs comprise a group of more than 50 histological subtypes, but in the current study, only the three most common subtypes were included in the splicing event analysis. Third, we assessed potential relationships between AS events and genetic alterations via a simple Spearman’s rank correlation test, which does not have the statistical power to understand the intrinsic mechanism of the influence of alternative splicing; the biological roles of the splicing events require further validation. Fifth, the correlation analyses conducted to link the expression of survival-associated splicing factors and AS events was a preliminary exploration of their surface relationship; more functional research is needed to uncover the underlying mechanism. Finally, we utilized classical splicing factors in our study; owing to the deficiency of protein-level data for these splicing factors, only their mRNA levels were analyzed. Because other RNA-binding proteins may also have an effect on splicing events, the relationship between splicing events and the complete repertoire of RNA-binding proteins in the human genome should be studied. Further in-depth analyses of alternative RNA splicing could provide new insights into the mechanisms of oncogenesis and indicate novel avenues for cancer therapy.

## Data Availability

The datasets used or analyzed during the current study are available from the corresponding author upon reasonable request.

## Abbreviations

STSs: soft tissue sarcomas
OS: overall survival
LPS: liposarcoma
LMS: leiomyosarcoma
UPS: undifferentiated pleomorphic sarcoma
AS: alternative splicing
TCGA: The Cancer Genome Atlas
PSI: percent spliced in
AAs: alternate acceptors
ADs: alternate donors
APs: alternate promoters
ATs: alternate terminators
ESs: exon skips
MEs: mutually exclusive exons
RIs: retained introns
ROC: receiver-operator characteristic
CNA: copy number alteration
RMS: rhabdomyosarcoma

## References

[1] Massarweh NN, Dickson PV, Anaya DA (2015). Soft tissue sarcomas: staging principles and prognostic nomograms. J Surg Oncol.

[2] Cormier JN, Pollock RE (2004). Soft tissue sarcomas. CA Cancer J Clin.

[3] Byerly S, Chopra S, Nassif NA (2016). The role of margins in extremity soft tissue sarcoma. J Surg Oncol.

[4] Thomas C, Movva S (2016). Eribulin in the management of inoperable soft-tissue sarcoma: patient selection and survival. Oncotargets Ther.

[5] Sarcoma Committee of Chinese Anti-Cancer A, Chinese Society of Clinical O (2016). Chinese expert consensus on diagnosis and treatment of soft tissue sarcomas (Version 2015). Chin J Oncol.

[6] Siegel RL, Miller KD, Jemal A (2018). Cancer statistics, 2018. CA Cancer J Clin.

[7] Kotilingam D, Lev DC, Lazar AJ, Pollock RE (2006). Staging soft tissue sarcoma: evolution and change. CA Cancer J Clin..

[8] Pollino S, Benassi MS, Pazzaglia L (2018). Prognostic role of XTP1/DEPDC1B and SDP35/DEPDC1A in high grade soft-tissue sarcomas. Histol Histopathol.

[9] Xie J, Lin D, Lee DH (2017). Copy number analysis identifies tumor suppressive lncRNAs in human osteosarcoma. Int J Oncol.

[10] Rello-Varona S, Tirado OM (2017). DNA methylation profiling opens a new phase in the search of targeted therapy against Ewing sarcoma. Pharmacogenomics..

[11] Feng H, Qin Z, Zhang X (2013). Opportunities and methods for studying alternative splicing in cancer with RNA-Seq. Cancer Lett.

[12] Bisognin A, Pizzini S, Perilli L (2014). An integrative framework identifies alternative splicing events in colorectal cancer development. Mol Oncol.

[13] Oltean S, Bates DO (2014). Hallmarks of alternative splicing in cancer. Oncogene.

[14] Matsushita K, Itoga S, Nishimura M, Furuta K, Nomura F (2015). Alternative splicing detection as biomarker candidates for cancer diagnosis and treatment with establishment of clinical biobank in Chiba University. Jpn J Clin Pathol.

[15] Chen J, Weiss WA (2015). Alternative splicing in cancer: implications for biology and therapy. Oncogene.

[16] Kornblihtt AR, Schor IE, Allo M, Dujardin G, Petrillo E, Munoz MJ (2013). Alternative splicing: a pivotal step between eukaryotic transcription and translation. Nat Rev Mol Cell Biol.

[17] Mazaki-Tovi S, Tarca AL, Vaisbuch E (2016). Characterization of visceral and subcutaneous adipose tissue transcriptome in pregnant women with and without spontaneous labor at term: implication of alternative splicing in the metabolic adaptations of adipose tissue to parturition. J Perinat Med.

[18] Baty F, Klingbiel D, Zappa F, Brutsche M (2015). High-throughput alternative splicing detection using dually constrained correspondence analysis (DCCA). J Biomed Inform.

[19] de Miguel FJ, Sharma RD, Pajares MJ, Montuenga LM, Rubio A, Pio R (2014). Identification of alternative splicing events regulated by the oncogenic factor SRSF1 in lung cancer. Can Res.

[20] Padgett RA (2012). New connections between splicing and human disease. Trends Genet.

[21] Balbinot C, Vanier M, Armant O (2017). Fine-tuning and autoregulation of the intestinal determinant and tumor suppressor homeobox gene CDX2 by alternative splicing. Cell Death Differ.

[22] Salton M, Kasprzak WK, Voss T, Shapiro BA, Poulikakos PI, Misteli T (2015). Inhibition of vemurafenib-resistant melanoma by interference with pre-mRNA splicing. Nat Commun.

[23] Sveen A, Kilpinen S, Ruusulehto A, Lothe RA, Skotheim RI (2016). Aberrant RNA splicing in cancer; expression changes and driver mutations of splicing factor genes. Oncogene.

[24] Li Y, Sun N, Lu Z (2017). Prognostic alternative mRNA splicing signature in non-small cell lung cancer. Cancer Lett.

[25] Zhu J, Chen Z, Yong L (2018). Systematic profiling of alternative splicing signature reveals prognostic predictor for ovarian cancer. Gynecol Oncol.

[26] He RQ, Zhou XG, Yi QY (2018). Prognostic signature of alternative splicing events in bladder urothelial carcinoma based on Spliceseq data from 317 cases. Cell Physiol Biochem.

[27] Lin P, He RQ, Ma FC (2018). Systematic analysis of survival-associated alternative splicing signatures in gastrointestinal pan-adenocarcinomas. EBioMedicine..

[28] Griffith M, Griffith OL, Mwenifumbo J (2010). Alternative expression analysis by RNA sequencing. Nat Methods.

[29] Ryan MC, Cleland J, Kim R, Wong WC, Weinstein JN (2012). SpliceSeq: a resource for analysis and visualization of RNA-Seq data on alternative splicing and its functional impacts. Bioinformatics.

[30] Lex A, Gehlenborg N, Strobelt H, Vuillemot R, Pfister H (2014). UpSet: visualization of intersecting sets. IEEE Trans Vis Comput Graph.

[31] Gao J, Aksoy BA, Dogrusoz U (2013). Integrative analysis of complex cancer genomics and clinical profiles using the cBioPortal. Sci Signal.

[32] Piva F, Giulietti M, Burini AB, Principato G (2012). SpliceAid 2: a database of human splicing factors expression data and RNA target motifs. Hum Mutat.

[33] Tselykh TV, Roos C, Heino TI (2005). The mitochondrial ribosome-specific MrpL55 protein is essential in *Drosophila* and dynamically required during development. Exp Cell Res.

[34] Liu S, Cheng C (2013). Alternative RNA splicing and cancer. Wiley Interdiscip Rev RNA..

[35] Brosseau JP, Lucier JF, Nwilati H (2014). Tumor microenvironment-associated modifications of alternative splicing. RNA.

[36] Busch A, Hertel KJ (2012). Evolution of SR protein and hnRNP splicing regulatory factors. Wiley Interdiscip Rev RNA..

[37] Hahn CN, Venugopal P, Scott HS, Hiwase DK (2015). Splice factor mutations and alternative splicing as drivers of hematopoietic malignancy. Immunol Rev.

[38] Paronetto MP, Passacantilli I, Sette C (2016). Alternative splicing and cell survival: from tissue homeostasis to disease. Cell Death Differ.

[39] Stricker TP, Brown CD, Bandlamudi C (2017). Robust stratification of breast cancer subtypes using differential patterns of transcript isoform expression. PLoS Genet.

[40] Xu Y, Gao XD, Lee JH (2014). Cell type-restricted activity of hnRNPM promotes breast cancer metastasis via regulating alternative splicing. Genes Dev.

[41] Sosulski A, Horn H, Zhang L (2016). CD44 splice variant v8-10 as a marker of serous ovarian cancer prognosis. PLoS ONE.

[42] Raso-Barnett L, Banky B, Barbai T, Becsagh P, Timar J, Raso E (2013). Demonstration of a melanoma-specific CD44 alternative splicing pattern that remains qualitatively stable, but shows quantitative changes during tumour progression. PLoS ONE.

[43] Zeilstra J, Joosten SP, van Andel H (2014). Stem cell CD44v isoforms promote intestinal cancer formation in Apc(min) mice downstream of Wnt signaling. Oncogene.

[44] Zhao YJ, Han HZ, Liang Y, Shi CZ, Zhu QC, Yang J (2015). Alternative splicing of VEGFA, APP and NUMB genes in colorectal cancer. World J Gastroenterol.

[45] Jacob AG, O’Brien D, Singh RK (2013). Stress-induced isoforms of MDM2 and MDM4 correlate with high-grade disease and an altered splicing network in pediatric rhabdomyosarcoma. Neoplasia..

[46] Zhang D, Duan Y, Cun J (2019). Identification of prognostic alternative splicing signature in breast carcinoma. Front Genet..

[47] Jung H, Lee D, Lee J (2015). Intron retention is a widespread mechanism of tumor-suppressor inactivation. Nat Genet.

[48] Diederichs S, Bartsch L, Berkmann JC (2016). The dark matter of the cancer genome: aberrations in regulatory elements, untranslated regions, splice sites, non-coding RNA and synonymous mutations. EMBO Mol Med.

[49] Barash Y, Calarco JA, Gao W (2010). Deciphering the splicing code. Nature.

[50] Sterne-Weiler T, Sanford JR (2014). Exon identity crisis: disease-causing mutations that disrupt the splicing code. Genome Biol.

[51] Singh S, Narayanan SP, Biswas K (2017). Intragenic DNA methylation and BORIS-mediated cancer-specific splicing contribute to the Warburg effect. Proc Natl Acad Sci USA.

[52] Yuan H, Li N, Fu D (2017). Histone methyltransferase SETD2 modulates alternative splicing to inhibit intestinal tumorigenesis. J Clin Investig.

